# Gynecological and Obstetrical Society of Baltimore

**Published:** 1891-03

**Authors:** William S. Gardner

**Affiliations:** Secretary; 410 Hanover street


### GYNECOLOGICAL AND OBSTETRICAL SOCIETY OF BALTIMORE.

### JANUARY MEETING.

The President, Dr. Henry M. Wilson, in the chair.

Dr. W. P. Chunn related an instance of apparent growth of the placenta after labor.

The patient was 28 years old, and had been married five years. She had had no children at full term, but had had three miscarriages. The first and second miscarriage occurred at about the fourth month of gestation. The last miscarriage occurred May 10th, 1890. She had missed one period and believed herself to be about six weeks pregnant. On the 10th of May she began to have bearing down pains and hemorrhage, with the expulsion of blood clots, lasting some three or four days. Then the pain subsided, the hemorrhage ceased, and I regarded the uterus as empty. On the 12th of June, however, she was again seized with violent pains, and during the night was delivered of placental mass larger than a man's fist, which I saw the next morning. The patient, as well as myself, was surprised. The fetus was searched for but no sign of it found.

Dr. Thos. A. Ashby :—I have seen a somewhat similar case. The patient began to have hemorrhage about the sixth week of gestation, but I was called in four weeks subsequently, and she was then in the act of throwing off the fetus. At the time of its removal the fetus was apparently at the 6th or 7th week of gestation, and partly decomposed. The placenta was not affected by decomposition. Before I saw her she had been going around bleeding from this cause, and was not aware that she was about to abort. She had had five miscarriages between the sixth and eighth week in twenty-eight months, so she stated.

Dr. G. W. Miltenberger : I have known the whole ovum to be retained for months after death of fetus. In a recent case the contents of the uterus were not thrown off till full term, though the fetus was dead at the third month.

I can not understand the growth of the placenta in utero after the death of the child; but I can conceive of the growth of the placenta outside the uterus, on account of the peculiar relation of the blood vessels.

Dr. L. E. Neale: I think it is very unfortunate that the specimen is not presented.

The placenta is not developed at the sixth week of pregnancy. The conditions in extra uterine pregnancy are very different from those in intra-uterine pregnancy, and what is true of one regarding placental development is not true of the other, and states his belief that the case was one of ordinary abortion (not miscarriage) with escape of the embryo, and more or less complete intention of the sac, chiefly chorian, and might have been removed at the time with a curette.

Dr. L. E. Neale read a paper upon "The Indications for Caesarean Section."

This paper is intended to stimulate interest in, and discussion of the subject caesarean section vs. craniotomy on the living child, upon which subject a series of papers will be presented by the members of the Society.

Craniotomy upon the living fetus is believed justifiable, but only as a dire necessity, not as an elective procedure, and should not be resorted to where there is a reasonable probability of success by the section and the uncoerced consent of the mother can be obtained.

No man is compelled to do craniotomy upon the living fetus solely upon the choice of the patient or her friends.

In answer to the question, "what would you do if the patient were your wife, your sister or a near relative? he believed practically this must be a matter for each man's conscience, over which no dogmatic rule of science can or should have sway. If seen early enough, the indication of premature labor at the 32d–34th week by the method of Krause was a very strong antagonist to craniotomy upon the living fetus. The range for this operation should not extend to a conjugata vera below 2 3-4 inches, or to one above 3 1-2 inches.

The indications for the conservative section included all insurmountable obstruction to the delivery of the living and viable child per vias naturales. They include tumors, pelvic exudations, hypertrophic elongation of the cervix, cicatrixes, stenosis, tetanusuter, falliform, uterine contractions, &c. He believed general opinion placed the limit for the absolute indication of a conjugata vera at 1 1-2 inches, and the relative indication extended from that point up to an undetermined conjugata vera measurement, and included many other conditions besides pelvic contractions. Other things being favorable, a 2 1-2 inch or 6.25 conjugata vera (Harris) 3 inch—7.5 cm. conjugata vera (Lusk) called for section rather than craniotomy, but be warned against relying entirely upon pelvinetry in the relative indication. In contracted pelvis, he preferred verrion to forceps when both were practicable.

The relative size of the head, its resistance—the past history—the uncoerced consent—the general condition and surroundings of the patient, &c., were all important factors in the relative indication.

The life of the child was not "purely impersonal and scientific," but eminently personal and practical, and he believed the mother should run a reasonable risk in its interest. The life saving of craniotomy could never be as great as that of C. S. for it started with a necessary mortality of 50 per cent., or half the lives at stake. He accepted Carl Bann's rules for the relative indication.

Craniotomy was safer for the mother than section, but piece meal extraction was equally, if not more dangerous—ex 92. conjugata vera 2 1-2 inches 6.25 cm. or less.

If coservative delivery p. v. n. had been attempted and fail ed, this was a strong point in favor of craniotomy, and against the section under these increased dangers.

He strongly deprecated conservative tampering and then resorting to the section, many lives had been thus sacrificed. If we desired success we must make the section an elective oper ation and not a procedure of dire necessity.

Dr. Miltenberger: With regard to the paper of Dr. Naeles,' confined as it is to the indications for the Caesarean section, there is nothing which I would controvert.

Under the absolute or positive indications as laid down, there can be no question.

The confusion and discrepancy of opinion have risen from want of definiteness and clearness as to the relative indications.

If we take the statistics of craniotomy generally, including all cases, we get no positive resulting data to guide us.

When the pelvis is so contractive as to necessitate the piece meal extraction of the fetus, it is recognized as the most serious of obstetric operations, and more dangerous than Caesarean section. Where, on the other hand, craniotomy alone is required, the operation is simple, and the danger to the mother in proper hands, should not be greater, than from the application of the forceps. In any individual experience on my own patients, I have been obliged to resort to craniotomy but twice in 50 years. The mothers all recovered.

Now it is just in this latter class that the doubt arises.

The smallest conjugata vera diameter through which a living child has been expelled is 3 inches, or as has been claimed, 2 3-4 inches, but with this we can not expect to save the child through the natural passages. But whether with this or a little more available space, we must recognize the prime and absolute importance, as the doctor states, of pelvimetry, and to this its thorough practical study and application must we mainly look for increased certainty. Especially does this hold as sectional pelvimetry, the best instrument, by far, being the hand of the obstetrist.

Now while it is true, the measure here of the conjugata vera by the finger may not be perfectly accurate, and we require also to learn the available space in the transverse diameter, yet with care, it sufficiently approximates the truth for our purpose.

When the practical obstetrist meets with a case of dystocia, from this cause, by internal measurement he satisfies himself as far as possible, he has three inches of available space in the conjugata vera, or even above this, without a full knowledge of the size of the fetal head, he naturally applies the forceps or proceeds to turn, and not improperly; but if he fails, he has already violated the first fundamental law in cæsarotomy, to resort at first to the knife without any previous operative manipulation, if such manipulation has been at all prolonged, the choice is not between craniotomy and caesarian section, but between craniotomy and a Porro.

Fortunately, pelvis contracted to this extent are rare in this country, particularly in the higher walks of life.

The operation of caesarotomy is, in itself, sufficiently simple and the modern section is undoubtedly one of the greatest advances in modern obstetrics, while its success constitutes a brilliant epoch in our recent history. In the hands of those skilled in its technique, and taught and trained by experience, there is every reason to trust and believe that the modern Saenger section will extend still further its successes, and that as the operator gains tact and knowledge with every case with which he deals, and as a part of the success must depend upon his absolute command of his patient and her surroundings, it is most likely the old picture will be reversed, and with our septic and antiseptic precautions, hospitals will offer a smaller rate of mortality than private practice.

I do not hesitate to say that I would prefer for my own wife, as the safer for her, craniotomy to caesarian section in such a case, and am therefore bound to extend to others, my patients. I am, therefore, forced to the opinion that caesarian section will not completely supplant the old operation, and that there still remains a field, although markedly limited, for craniotomy on the living child.

Dr. J. Whitridge Williams: I am sure that we are all greatly indebted to Dr. Neale for the very clear manner in which he has set forth the indication for the operation, and I almost entirely agree with him.

The absolute indication I would place at 5-5 1-2 cm., or three inches, and the upper limit for the relative indication at 7 1-2 cm., or three inches. Within these limits, unless the child be abnormally small, there should be no question as to the use of forceps; and the question to be decided is, whether craniotomy or caesarian section should be done.

Theoretically, I might choose the section in all cases that appeared favorable; but practically, I might waive my theory in the case of a primipara who had not been examined previous to labor.

But if I performed craniotomy under these circumstances, I

would warn her that in becoming pregnant again, she would take the responsibility of the child's life upon herself, and that I would refuse to perforate in subsequent pregnancies.

The mortality of the operation need not deter us, for Munchmeyer has lately reported the latest statistics of Leopold, in which he reports 28 Seanger operations, with the loss of three mothers and one child, and 9 Porro operations with no maternal deaths.

Dr. B. B. Browne: I had a case recently upon which I did caesarian section. The woman was 27 years of age. She had had one child. Her labor was two years ago, when she had convulsions, and a craniotomy was done. As a result of injury received at this time, the vagina and uterus sloughed, and there was complete atresia of the vagina. This atresia was afterward opened up, and she became pregnant. The vagina was contracted by cicatrical bands, and an opening could be felt in the side of the cervix, but to the left of the opening was a cup-shaped cavity which might have been the old cervix.

She was not sure of the time of impregnation. She was swollen, and her urine was solidified with albumin upon heating. Labor pains began December 20th, and continued for one or two days, but there was no dilatation. She came to the hospital December 22d. She had severe uterine contractions that day, and came for the purpose of having caesarean section done. But next day the pains had all gone. On the night of January 1st the water broke, and severe pains began. The cicatrical bands about the cervix were cut and Elliot's forceps were introduced. Both blades of Tornier's forceps could not be gotten on. After several efforts, I concluded that she could not be delivered in that way. In the morning the fetal heart was distinct, in the afternoon it was feeble.

The section was made without difficulty. The placenta was attached in front. The child could not be resuscitated. The placenta was readily detached and the uterus was cleaned out and closed by the Seanger method.

The operation was done on Friday, and the patient did well until the following Tuesday, when she sank rapidly, and died in a few hours.

The woman had grave kidney disease, and had little chance of recovery on that account.

In this case several things are to be considered:

1st. The woman was perfectly willing for the operation.

2d. Her life, from the condition of her kidneys, was not insurable, and the child had a good chance of living.

3d. She had much difficulty in the former craniotomy, and barely escaped with her life.

Dr. Ashby: I have had the good fortune to witness two caesarean sections. One, the case of Dr. Jay, of this city, several years ago, and the recent case reported by Dr. Browne. I was impressed with the ease with which the operation can be done. Its mechanical execution is certainly much less difficult than is necessitated by many intra-abdominal operations. Hemorrhage is easily controlled, and the closure of the uterine wound is not a difficult undertaking.

In the case of Dr. Jay, the mother made a prompt recovery, and the child perished simply because of the unavoidable delay which was experienced before an attempt at its removal was made. Its death had, in my opinion, no relation to the operation, but to causes which antedated the section. I am convinced in the case of Dr. Browne the child could have been saved had no other method of delivery been attempted. The section, I think, bore no relation to its death.

The operation of the future will be approached without delay, and before other methods of delivery have been employed.

The important indication for the operation rests upon careful pelvic measurements and discrimination in advance of any obstetric interference of the impossibility of delivery by version or forceps. If this is done, the section will be approached under its most favorable aspects, and its results will be far more satisfactory.

I agree with Dr. Miltenberger in that personally I would prefer craniotomy, if the patient were a member of my own family, but upon scientific grounds I would not hesitate to operate did my patient and her friends elect this procedure, having satisfied my own mind that a living child could not be born in any other way.

Dr. Neale: As no points were raised against the paper, I have nothing to say in its defence.

I did examine Dr. Browne's case, and told him in my opinion it was no case for the section.

The chief obstruction was in the soft parts, that of the pelvis was very slight, if any. I thought it possible to deliver the child alive p. v. n., but was sure it could be readily extracted after craniotomy. Owing to the kidney complication, the mother was in a most unfavorable condition for the section, and for that matter, the child also, therefore I advised against this operation. WILLIAM S. GARDNER,

410 Hanover street. Secretary.

